# An increase in global daily precipitation records in response to global warming based on reanalysis and observations

**DOI:** 10.12688/openreseurope.17674.1

**Published:** 2024-06-10

**Authors:** James Ciarlo', Filippo Giorgi

**Affiliations:** 1 Abdus Salam International Centre for Theoretical Physics (ICTP), Trieste, Italy; 2 University of Malta, Msida, Malta

**Keywords:** extreme events, precipitation, precipitation change, precipitation records

## Abstract

Understanding trends in extreme precipitation events in the context of global warming is critical for assessing climate change impacts. This study employs a novel methodology developed by Giorgi and Ciarlo (2022) to analyze record-breaking daily precipitation events from 1980 to 2020, utilizing three reanalysis products (ERA5, MERRA-2, and JRA-55) and one global observation dataset (MSWEP). Our results indicate a consistent and statistically significant increase in record-breaking precipitation events globally, with variations across different latitude bands and between land and ocean areas. This trend is evident in all datasets, with the most substantial increases observed over oceans in ERA5 and over land in JRA and MERRA. Notably, the Southern Hemisphere shows mixed results, with some regions displaying negative trends. This study highlights the increasing frequency of extreme precipitation events, supporting the hypothesis of intensified hydrological cycles under a warming climate. Our findings enhance understanding of precipitation extremes and underscore the importance of regional analyses in climate impact studies. Future work could extend these findings to formal attribution studies linking observed trends directly to anthropogenic climate change.

## Plain Language Summary

In recent decades, observations have shown changes in how often and how intensely it rains, which can be linked to global warming. Our study analyses record-breaking rainfall events, i.e. days when rainfall reaches unprecedented highs, in different observational and reanalysis records for the last 40 years. We use a new method to compare daily rainfall records with the values that would be expected in stable climate conditions, i.e. without warming.

Our findings show that extreme rainfall events have become more frequent around the world. This trend is predominant across various latitudinal regions and over oceans and land, though there are some differences depending on the location. Notably, the increase in record rainfall events is more consistent across the oceans than the continental regions, with some of the latter showing negative trends in the southern hemisphere. This conclusion has important implications for how we prepare for and manage flooding and other related natural disasters in the future.

## Introduction

The issue of whether precipitation extremes have significantly increased over the last decades, possibly due to climate warming, has been a controversial one within the global change debate ( Coppola *et al.*, 2021; Giorgi *et al.*, 2011; Giorgi *et al.*, 2014a; Giorgi *et al.*, 2014b; Giorgi *et al.*, 2019; Held & Soden 2016; Martinkova & Kysley, 2020; Sillmann *et al.*, 2013; Trenberth *et al.*, 2003). One of the difficulties in this regard is that precipitation extremes are by definition rare and thus are highly variable in space and time, so that significant trends are difficult to identify. Moreover, different metrics can be used to define extremes (e.g., the 95 ^th^ or 99 ^th^ percentiles of the precipitation intensity distribution, or the maximum daily rain intensity within a year) and the results may depend on the metrics utilized. Chapter 11 of the WGI Sixth Assessment Report ( Seneviratne *et al.*, 2021, and references therein) presents a comprehensive overview and assessment of studies of observed trends in precipitation extremes during past decades and finds that significant positive trends are observed over most continental regions, and that areas (or the number of stations) of significant increase are much larger and more widespread across continents than areas of decrease.

In this brief report, we want to add to this discussion by using a method recently developed by Giorgi and Ciarlo (2022) (GC22) and Belleri *et al.* (2023) (BE23) to identify record-breaking events (or more simply “records”) in daily precipitation time series and comparing the number of detected events in the series with that theoretically expected in stationary climate conditions, i.e. conditions of no warming (thereafter referred to as “reference” value). This theoretical estimate is equal to 1/ *n*, where *n* is the *n*th year after the beginning of the time series, if no significant autocorrelation is expected in the time series, which is the case when comparing daily precipitation for different years. The method of GC22 is an extension of an analogous method used to detect increases in the number of daily temperature records throughout the year compared to the reference value as a signature of climate warming (e.g., Elguindi *et al.*, 2013; Jones, 2016; Meehl *et al.*, 2016; Powell & Delage, 2019).

We calculate the number of precipitation records globally and in different latitude bands for the period 1980–2020 in three reanalysis products and one global observation product to identify whether this number is significantly different from what expected theoretically in stationary climate conditions.

## Methods

The extension proposed by GC22 and BE23 aims to address the problem of the large number of 0-values (dry days) present in daily precipitation time series, and is based on the detection of records in maximum daily precipitation in a 30-day moving window across the year, rather than the precipitation value at a given day of the year. In other words, rather than comparing precipitation on a given day of the year, say April 15, to all corresponding values in previous years, the comparison is carried out using the maximum daily value within the 30-day period centred around April 15, i.e., April 1–30. The procedure is then repeated for all 30-day moving windows throughout the year, whose number is 337 (instead of 365).

As a measure of deviations from stationary conditions, GC22 and BE23 use the Ratio of Actual (detected)-to-Reference number of records, a metric referred to as RAtR. A value of RAtR greater (smaller) than 1 thus indicates a greater (smaller) number of detected records compared to the reference value, and is thus indicative of trends in records (as a measure of extremes) within the time series analysed with respect to stationary conditions. In addition, as discussed by GC22 and BE23, the method is best applied at the regional scale, i.e., by aggregating the numbers of records over a given region, rather than considering individual grid points or stations, in order to minimize the occurrence of noise and obtain a more robust signal. The reader is referred to GC22 and BE23 for more detail on the method.

GC22 applied this methodology to the E-OBS observations dataset, the ERA5 reanalysis and some regional climate projections over the European region carried out under the EURO-CORDEX initiative ( Jacob *et al.*, 2020), and found that an increase in RAtR is a consistent signal associated with warming. In particular, they also found a positive trend of RAtR aggregated over the European region in the E-OBS dataset from 1950 to 2020. BE23 then extended the same analysis to the 9 continental scale domains used in the CORDEX-CORE project ( Giorgi *et al.*, 2022) and assessed different regional observation datasets from 1950 to 2020, three reanalysis products and the CORDEX-CORE projections. They confirmed that an increasing trend in regionally aggregated RAtR is a prevailing signal associated with warming, particularly in the projections. However, they also found a substantial variability and some inconsistencies of RAtR trend sign (presented as a change in RAtR per decade) across the different observation datasets. They thus concluded that their regional analysis did not provide fully consistent indications concerning RAtR trends in the observational records for the last decades, although there was a prevalence of positive trends.

In this brief report, we extend the analysis of BE23 globally, focusing only on the question whether there are trends in the daily precipitation RAtR values, as obtained with the methodology of GC22, during the last decades. Specifically, we focus on the period 1980–2020 and use three reanalysis products, ERA5 ( Hersbach *et al.*, 2020), MERRA-2 ( Gelaro *et al.*, 2017, hereafter referred to as MERRA) and JRA-55 ( Kobayashi *et al.*, 2015, hereafter referred to as JRA) and one global observation dataset, MSWEP ( Schneider *et al.*, 2013), all including daily precipitation rate within the selected analysis period.

The GC22 methodology was applied to these datasets to assess the global signal, as well as the sperate components of land and ocean, through the application of appropriate masks. The analysis also included data sub-sets differentiated according to latitude bands, specifically, tropics (Equator to 30°N/S), mid-latitudes (30–60°N/S) and polar regions (60–90°N/S), for land and ocean. This allowed for a holistic interpretation of the results whilst assessing the source of global signals.

## Results

**Figure 1.**
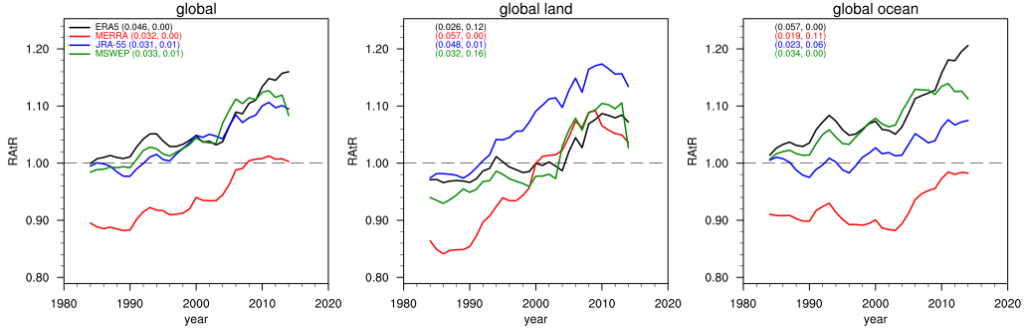
10-year running mean of the RAtR values for the different precipitation datasets for the period 1980–2020. The coefficient and p-values of the trend lines (units of 1/decade) are also reported for each dataset (first and second number in parentheses, respectively).

Figure 1 shows the RAtR values aggregated globally and over global-land and global-ocean areas in the four products for the period 1980–2020. It can be seen that in all cases the trend in RAtR is positive, and for most cases it is statistically significant at the 90% confidence level. In all but 4 cases the statistical significance is at the 95% confidence level. Over the 40-year period, the increase in RAtR ranges between 10 and 15%, with larger values (up to 20%) for ERA5-Ocean, JRA-Land, and MERRA-land. Note that in the case of MERRA the value of RAtR is lower than 1 for the first few decades of the time series, although still with a positive trend. This may be attributed to the sensitivity of this calculation to the initial year of the series, which evidently, for MERRA, had a relatively high number of intense events. Therefore, in our analysis it is more important to assess the RAtR trends than the actual values.

**Figure 2.**
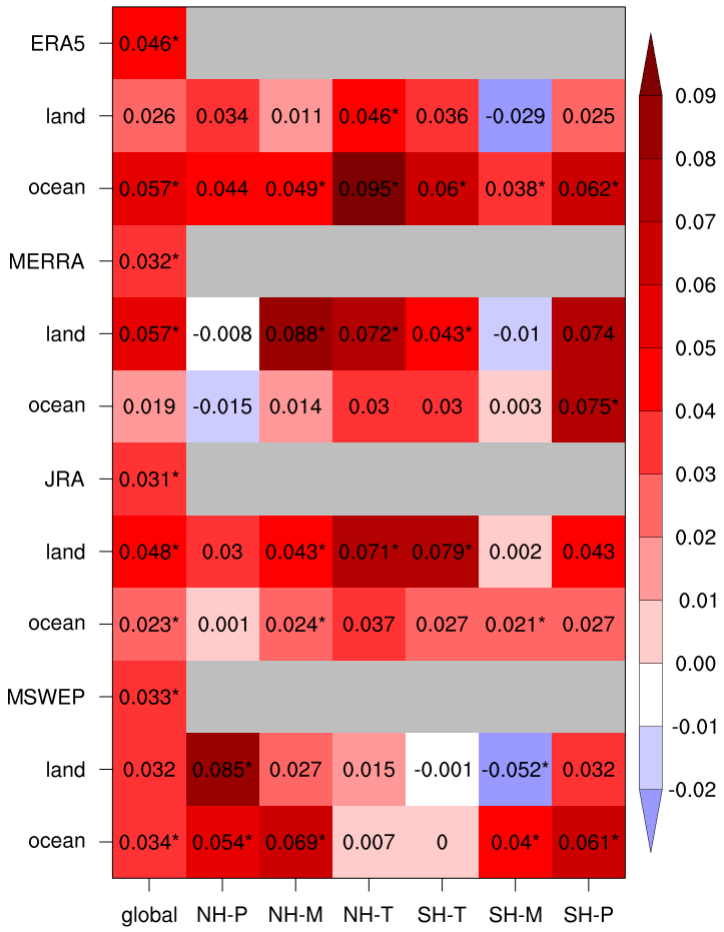
Linear trend values (units of 1/decade) of the 10-year running average of the RAtR values for each global dataset, showing also the land-only and ocean-only values. For the latter two, the RAtR values are presented as global, and according to the six global bands defined in Methods. Asterisks indicates that the trend is statistically significant at the 90% confidence level.

The entire set of 1980–2020 RAtR trend results are summarized in Figure 2, which also includes values aggregated over different latitude bands, i.e. tropics (Equator to 30°N/S), mid-latitudes (30–60°N/S) and polar regions (60–90°N/S), for land & ocean and the land/ocean only portions of the areas. The statistical significance of the trends in the figure is calculated at the 90% confidence level.

As already seen, at the global scale, all products show positive and statistically significant RAtR trends, the largest in ERA5 over ocean, and JRA and MERRA over land. Moving to the different latitude bands, we find a strong predominance of positive trends, especially in the three reanalysis products. Over the Northern Hemisphere (NH) latitude bands only two cases of negative trend are found (polar-land and ocean in MERRA, but not statistically significant), while in 11 out of 22 positive trend cases, the trend itself is statistically significant. In the Southern Hemisphere (SH), the results are more mixed, with 4 cases of negative trends (one statistically significant). In this regard, of interest is the fact that all products show negative trends over SH mid-latitude land areas, which comprise southern South America, the southern tip of the African continent and southern Australia. We also note that for MSWEP in two out of six SH cases we find negative trends, while the three reanalysis products show positive trends everywhere, except the tropical SH ocean, which has a negligible trend. Thus, over the SH the reanalysis products are more in agreement with each other than with the observational product.

## Conclusions

In summary, our analysis of a global observation dataset and three reanalysis products supports the conclusion that extreme daily precipitation events have been increasing during recent decades compared to what is expected from stationary climate conditions, where we use as metric the number of record-breaking cases of maximum daily precipitation within 30-day moving windows. We compared our approach to the use of separate 30-day periods in the analysis, e.g., separate months (January, February, March, etc.) and to the use of 15-day long moving windows, and we found results qualitatively in line with those of Figure 1 and Figure 2. We also recognize that the occurrence of local record-braking events may depend on shifts in precipitation patterns, but this effect is minimized by the global and large regional analysis. Our analysis is limited to a diagnostic assessment of data over the last decades and is not intended to provide a formal attribution study of the observed trends to the global warming which occurred during the same decades. This, however, could be a next step application of our methodology.

## Data Availability

The ERA5 dataset, produced on the ECMWF high-performance computing facility, is available on the CDS cloud server [Accessed: 01/02/2022] ( https://doi.org/10.24381/cds.143582cf); MERRA is produced by NASA Global Modeling and Assimilation Office (GMAO) and available on Goddard Earth Sciences Data and Information Services Center website [Accessed: 07/05/2022] ( https://doi.org/10.5067/7MCPBJ41Y0K6); JRA-55 has been produced with the TL319 version of the Japan Meteorological Agency (JMA) operational data assimilation system [Accessed: 01/03/2022] ( https://doi.org/10.5065/D6HH6H41); MSWEP produced by Beck, Hylke & National Center for Atmospheric Research Staff (Eds). Last modified 2023-09-05 "The Climate Data Guide: Global high-resolution precipitation: MSWEP” [Accessed: 23/06/2022, https://climatedataguide.ucar.edu/climate-data/global-high-resolution-precipitation-mswep].

## Software availability

Source code (developed from the GC22 paper, and updated for the BE23 paper) available from: https://github.com/ciarloj/Climate-Records-processor

Archived software available from: https://doi.org/10.5281/zenodo.11243112 ( Ciarlo, 2024)

License: CC-BY-4.0
