# Supplementary material for: An increase in global daily precipitation records in response to global warming based on reanalysis and observations
Source: Open Res Eur. 2024 Jul 29;4:114. Originally published 2024 Jun 10. [Version 2] doi: 10.12688/openreseurope.17674.2 (PMC11220443; doi:10.12688/openreseurope.17674.2)
Supplement: Peer review history

# Reviewer report: Reviewer response for version 2

**DOI:** 10.21956/openreseurope.19825.r48174

**Published:** 2025-01-07

**Authors:** Fulden Batibeniz

**Affiliations:** 1 University of Bern Oeschger Centre for Climate Change Research, Bern, Canton of Bern, Switzerland; 2 Department of Environmental Systems Science, Institute for Atmospheric and Climate Science, Zurich, Switzerland

Despite the extensive literature on extreme indices and comprehensive coverage in the IPCC report, this study provides valuable new insights. With record-breaking events occurring more frequently as global mean temperatures rise, this study effectively demonstrates both how climate change influences the increase in precipitation extremes and the increasing frequency of record-breaking events. I recommend accepting the paper for indexing with minor revisions as noted below.

 1-In results section: Please clarify this sentence: "In all but 4 cases the statistical significance is at the 95% confidence level."

 2-In conclusion: Typo “ *record-braking”.*

 3-In conclusion: The following sentence needs clarification: "We also recognize that the occurrence of local record-braking events may depend on shifts in precipitation patterns, but this effect is minimized by the global and large regional analysis." Does this mean that averaging minimizes the effect of pattern shifts? Additionally, aren't precipitation pattern shifts also linked to climate change? Consider revising this sentence for clarity.

Is the study design appropriate and does the work have academic merit?

Yes

Is the work clearly and accurately presented and does it cite the current literature?

Yes

If applicable, is the statistical analysis and its interpretation appropriate?

Yes

Are all the source data underlying the results available to ensure full reproducibility?

Yes

Are the conclusions drawn adequately supported by the results?

Yes

Are sufficient details of methods and analysis provided to allow replication by others?

Yes

Reviewer Expertise:

Climate Modelling, Regional Earth System Modelling, Climate Variability and Change, Extremes, Compound events

I confirm that I have read this submission and believe that I have an appropriate level of expertise to confirm that it is of an acceptable scientific standard.

# Reviewer report: Reviewer response for version 2

**DOI:** 10.21956/openreseurope.19825.r48175

**Published:** 2024-12-12

**Authors:** Kalle Nordling

**Affiliations:** 1 Finnish Meteorological Institute, Helsinki, Finland

This article examines how precipitation records increase in observed climate period. based on 4 observational and reanalysis dataset. This article argues that extreme precipitation events increases when only looking record braking events.

 In abstract author states " Our results indicate a consistent and statistically significant increase in record-breaking precipitation events globally, " here it is unclear what does it mean by statistically significant and  how it was tested

 From text it is hard to understand how RAtR is calculated, can you ad equation for this?

 In figure 1, what tests provides the p value?

 The  article does not evaluate how well reanalysis datasets captures observed extreme precipitation.

Is the study design appropriate and does the work have academic merit?

Yes

Is the work clearly and accurately presented and does it cite the current literature?

Partly

If applicable, is the statistical analysis and its interpretation appropriate?

Partly

Are all the source data underlying the results available to ensure full reproducibility?

Yes

Are the conclusions drawn adequately supported by the results?

Partly

Are sufficient details of methods and analysis provided to allow replication by others?

Partly

Reviewer Expertise:

Climate modeling, climate  variability, extreme events, aerosols

I confirm that I have read this submission and believe that I have an appropriate level of expertise to confirm that it is of an acceptable scientific standard, however I have significant reservations, as outlined above.

# Response

**Published:** 2024-12-17

**Authors:** James Ciarlo

**Affiliations:** University of Malta, Msida, Malta

*We are very grateful to the time and suggestions of the reviewer. Below, we address the points raised:* *With regards to the statistical significance,* *for most cases it is statistically significant at the 90% confidence level. In all but 4 cases the statistical significance is at the 95% confidence level. This is extracted from the first paragraph of the Results section.* *The method used to provide the p-value is indicated in Belleri et al. (2023), where the paper specifies that this is calculated using the NCL package, which employs an ANOVA-based approach.* *The RAtR is defined as the “Ratio of Actual (detected)-to-Reference number of records” in the introduction of this brief report and detailed further in Belleri et al. (2023) where the term was first defined.* *The article does not evaluate the reanalysis datasets for extreme precipitation as this has already been covered extensively in other studies. Some of these studies are listed below: *

- *Lavers, D.A., Simmons, A., Vamborg, F. & Rodwell, M.J.(2022) An evaluation of ERA5 precipitation for climate monitoring. Quarterly Journal of the Royal Meteorological Society, 148(748) 3124–3137. Available from: https://doi.org/10.1002/qj.4351*
- *Lei X, et al. (2022). How Well Does the ERA5 Reanalysis Capture the Extreme Climate Events Over China? Part I: Extreme Precipitation. Frontiers in Environmental Science, 10. Doi: 10.3389/fenvs.2022.921658*
- *Ashouri, H., S. Sorooshian, K. Hsu, M. G. Bosilovich, J. Lee, M. F. Wehner, and A. Collow, 2016: Evaluation of NASA’s MERRA Precipitation Product in Reproducing the Observed Trend and Distribution of Extreme Precipitation Events in the United States. J. Hydrometeor., **17**, 693–711, https://doi.org/10.1175/JHM-D-15-0097.1.*
- *Savtchenko, A. K., G. Huffman, and B. Vollmer (2015), Assessment of precipitation anomalies in California using TRMM and MERRA data, J. Geophys. Res. Atmos., 120, 8206–8215, doi: 10.1002/2015JD023573.*
- *Shibuya, R., Y. Takayabu, and H. Kamahori, 2021: Dynamics of Widespread Extreme Precipitation Events and the Associated Large-Scale Environment Using AMeDAS and JRA-55 Data. J. Climate, **34**, 8955–8970, https://doi.org/10.1175/JCLI-D-21-0064.1.*

# Reviewer report: Reviewer response for version 1

**DOI:** 10.21956/openreseurope.19099.r41454

**Published:** 2024-07-02

**Authors:** Wei Li

**Affiliations:** 1 Nanjing University of Information Science and Technology, Nanjing, Jiangsu, China

This article employs a novel method to analyze trends in record-breaking daily precipitation over global land and oceans. It finds a significant increase in record-breaking extreme rainfall worldwide. I suggest that the document introduce the methods mentioned in the article, such as how RAtR is defined, since RAtR is a key metric in the study. Additionally, results for different latitude bands should be presented.

Is the study design appropriate and does the work have academic merit?

Yes

Is the work clearly and accurately presented and does it cite the current literature?

Yes

If applicable, is the statistical analysis and its interpretation appropriate?

Yes

Are all the source data underlying the results available to ensure full reproducibility?

Yes

Are the conclusions drawn adequately supported by the results?

Yes

Are sufficient details of methods and analysis provided to allow replication by others?

Partly

Reviewer Expertise:

Detection and attribution of extreme precipiltation at regional scale.

I confirm that I have read this submission and believe that I have an appropriate level of expertise to confirm that it is of an acceptable scientific standard.

# Response

**Published:** 2024-07-24

**Authors:** James Ciarlo

**Affiliations:** University of Malta, Msida, Malta

*We are very grateful to the time and suggestions of the reviewer. We have updated the text to clarify how the RAtR is defined. We did not however, provide new updates to the results for different latitude bands, as (assuming we did not misunderstand the reviewer’s comments) these are covered in Figure 2 and the adjoining discussions.*

# Reviewer report: Reviewer response for version 1

**DOI:** 10.21956/openreseurope.19099.r41453

**Published:** 2024-06-25

**Authors:** Cristian Lussana

**Affiliations:** 1 The Norwegian Meteorological Institute, Oslo, Norway

The manuscript describes statistical analysis methods applied to reanalyses and observational gridded datasets, aiming to investigate trends in the occurrence of extreme precipitation events. The topic is of great importance as it links variations in extreme event occurrences to global warming. The datasets considered in this study are recognized for their high quality in climate studies. Despite having their pros and cons, which are often documented by the producers, these datasets provide valuable information on climate change. The statistical analysis is well described by the authors, and the interpretation of results is appropriate. The conclusions are supported by the results.

 I have two comments:

 1. The authors may consider referring to Buschow (2024) to explain part of the differences found between ERA5 and the other datasets: Buschow, S. (2024). Tropical convection in ERA5 has partly shifted from parameterized to resolved. Quarterly Journal of the Royal Meteorological Society, 150(758), 436–446. Available from:(Buschow S et al 2024) [Ref 1]

 2. At the beginning of the Methods section, the authors may consider expanding the first paragraph by adding more details on the application of the method. For instance, it is not clear why they limit the analysis to 337 moving windows, as it seems feasible to use the previous 15 days also for the 1st of January. Is there a specific reason for this limitation?

Is the study design appropriate and does the work have academic merit?

Yes

Is the work clearly and accurately presented and does it cite the current literature?

Partly

If applicable, is the statistical analysis and its interpretation appropriate?

Yes

Are all the source data underlying the results available to ensure full reproducibility?

Yes

Are the conclusions drawn adequately supported by the results?

Yes

Are sufficient details of methods and analysis provided to allow replication by others?

Partly

Reviewer Expertise:

Climatology; Meteorology

I confirm that I have read this submission and believe that I have an appropriate level of expertise to confirm that it is of an acceptable scientific standard.

## References

[1] : Tropical convection in ERA5 has partly shifted from parameterized to resolved. *Quarterly Journal of the Royal Meteorological Society* .2024;150(758) : 10.1002/qj.4604 436-446 10.1002/qj.4604

# Response

**Published:** 2024-07-24

**Authors:** James Ciarlo

**Affiliations:** University of Malta, Msida, Malta

*We are very grateful to the time and suggestions of the reviewer. Here are our responses to your points:*

1. *We have included a comment making reference to the similar observations of Buschow (2024).  *
2. *We have limited the analysis to 337 moving windows in order to avoid overlap between different years. We have clarified this in the Methods section.*
